# Design and validation of an automated healthcare-integrated biobanking algorithm for identification of advanced chronic kidney disease

**DOI:** 10.1016/j.csbj.2025.10.061

**Published:** 2025-11-02

**Authors:** Claudia Fischer, Boris Betz, Johannes Stolp, Danny Ammon, André Scherag, Michael Kiehntopf

**Affiliations:** a Institute of Medical Statistics, Computer and Data Sciences (IMSID), Jena University Hospital – Friedrich Schiller University Jena, Jena, Germany; b Department of Clinical Chemistry and Laboratory Diagnostics and Integrated Biobank Jena (IBBJ), Jena University Hospital – Friedrich Schiller University Jena, Jena, Germany; c Data Integration Center, Jena University Hospital – Friedrich Schiller University Jena, Jena, Germany

**Keywords:** Healthcare-integrated biobanking, HIB algorithms, Chronic kidney disease, Silver-standard cohort, Electronic health records (EHR)

## Abstract

Healthcare-integrated biobanking (HIB) describes the collection of surplus samples from clinical routine and requires tailored algorithms for identification of adequate samples. However, identifying patients with specific conditions like chronic kidney disease (CKD) from heterogeneous real-world data remains challenging. This study develops and validates two HIB-specific algorithms for automated CKD identification based on electronic health records (EHR), enabling targeted sample collection and retrospective cohort assembly. Two logistic regression-based CKD algorithms were developed in an existing training cohort (n = 785) with high prevalence of CKD (48 %): the admissionHIB algorithm (based on laboratory values at admission) and the historyHIB algorithm (including additional previous hospital stays). The validation was carried out on patients of Jena University Hospital who gave informed consent to the Broad Consent of the Medical Informatics Initiative (MII) and were admitted between 01/2018 and 04/2020. The validation cohort was divided into a gold-standard cohort (n = 162) defined by manual chart review and a larger silver-standard cohort (n = 1075) generated using a validated algorithm from prior studies. The admissionHIB and historyHIB algorithms achieved F1-scores of 86 % and 91 %, respectively, in the training cohort. The validation cohort had a lower prevalence of CKD (approximately 12 %). Several automated review algorithms were evaluated in the gold-standard cohort, with the best-performing model (93 % recall, precision, and F1-score; 97 % accuracy) selected to generate the silver-standard cohort. Both HIB algorithms yielded F1-scores of 80 % (admissionHIB) and 78 % (historyHIB) in the gold-standard cohort, and 83 % and 80 %, respectively, in the silver-standard cohort. These findings demonstrate good performance of HIB-specific CKD algorithms across heterogeneous patient populations, establishing a reproducible framework combining real-world EHR data, patient consent infrastructure, and silver-standard validation.

## Graphical Abstract

**Figure:**
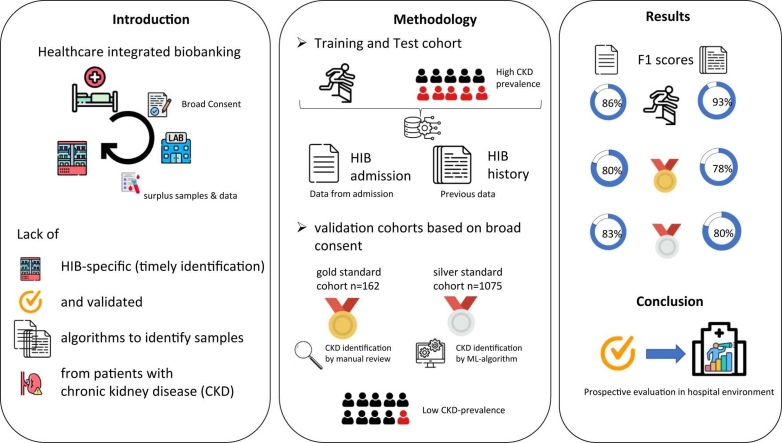

## 1 Introduction

Healthcare-integrated biobanking (HIB) allows collecting samples for well-phenotyped study cohorts in a clinical routine setting. This “live biobanking” approach utilizes residual material obtained after diagnostic routine has been performed [1]. The advantage of HIB, when efficiently integrated into a smart hospital’s information workflow, is the possibility to rapidly build large, quality-controlled cohorts of samples from a “real-world” hospitalized population under defined pre-analytical conditions.

Timely collection and correct interpretation of clinical phenotype data are the essential prerequisites for sample collection, as they determine which samples should be collected.

The early collection of samples for HIB increases the likelihood of biological integrity and clinical relevance of the samples. The impact of treatments, medical procedures and disease progressions, which occur during the hospital stay, is minimized, which can be essential for investigating disease mechanisms as well as identifying novel biomarkers and treatments where sample quality and timing of sample collection can have large impact on downstream analysis. In addition, traceability and standardization of processes are supported by early sample selection.

For the early identification of target samples HIB, phenotyping algorithms need to be integrated into the hospital information system (HIS).

In this study, we develop and validate HIB phenotyping algorithms for the use case of advanced chronic kidney disease (CKD). CKD is a major public health concern characterized by increasing prevalence and associated with high morbidity and mortality [2], [3]. Accurate identification of CKD is essential for clinical research, including clinical trials and biomarker validation. In this context, a particular challenge is to store samples from hospitalized patients with known kidney status in clinical biorepositories as part of HIB. At the time of sample selection and storage shortly after hospital admission, only a limited set of information/parameters regarding the patient is available. Although many CKD phenotyping algorithms have been published [4], to our knowledge, this specific key requirement for HIB has been hardly considered. Most existing algorithms rely on parameters that are not regularly available in HIS immediately at hospital admission. These include experimental or non-routine parameters such as retinal imaging [5], metabolite biomarkers [6], or renal biopsy results [7]. In addition, information about medical history or physical examination findings (e.g., appetite, pedal edema) [8] or about acute or chronic comorbidities (e.g., AKI, sepsis, diabetes, heart disease) [9] that evolve or are documented later during hospitalization are only available in HIS at a later stage of the hospital stay - or regarding discharge summaries after discharge.

CKD phenotyping algorithms are often either based on data from epidemiological studies or repositories (for example [10]) to optimize mathematical models or feature selection processes. If suggested as part of Clinical Decision Support Systems (CDSS) algorithms make use of electronic health record (EHR) data at a later point in time during the hospital stay not aiming at HIB (Table 1).

**Table 1:** Schematic summary of differences between phenotyping algorithms for Clinical Decision Support Systems (CDSS), for healthcare-integrated biobanking (HIB) or for retrospective epidemiological studies/registries respectively.

	Phenotyping algorithms in CDSS*	Phenotyping algorithms in HIB	Phenotyping algorithms in retrospective epidemiological studies / registries
purpose	Identifiation of patients at risk for application of therapy, increased surveillance	Collection of surplus biosamples from clinical routine for creation of well-characterized cohorts for future research	Retrospective analysis of patient/cohort subgroups with specific characteristics from real-word data and from study data
Time point of deployment during index hospital stay	During the course of hospital stay	Early to ensure high quality of the sample collection	After hospital stay
Availability of information	Usually much information from the EHR is available	Fractured information is available due to early use of the system	All information about the patient in EHR is available
Main metric	Focus on Recall so that no patient at risk is overlooked	Focus on Precision so that no false-positive samples are collected	Balance between recall and precision for best accurate division into subgroups
Setting of deployment	Integration into the hospital information system (HIS) to inform the clinician*	Integration into an intrainstitutional research platform that is connected to HIS to enable timely sample collection	Can be performed on an external platform when data can be transferred

* often such CDSS will fall under regulations such as the EU Medical device regulation

Another problem of phenotyping algorithms in general is the lack of a thorough validation [4], [11], [12]. This deficiency can be partly attributed to the time and resource-intensive nature of generating a gold-standard through manual review of the EHRs, as demonstrated by our group for CKD [13]. Furthermore, efforts to integrate algorithms enabling HIB are found even rarer. Potential reasons are the necessity to link the HIB phenotyping algorithms to data from the hospital information systems, which in turn implies limitations related to data quality and availability.

To address this lack of HIB-specific validated algorithms for CKD in this study we first designed phenotyping algorithms for advanced CKD tailored to the HIB setting. Second, we validated the HIB algorithms in a smaller gold-standard cohort created by manual review and a larger silver-standard cohort created by automated classification.

## 2 Material and methods

### 2.1 Study population

**Fig. 1:**
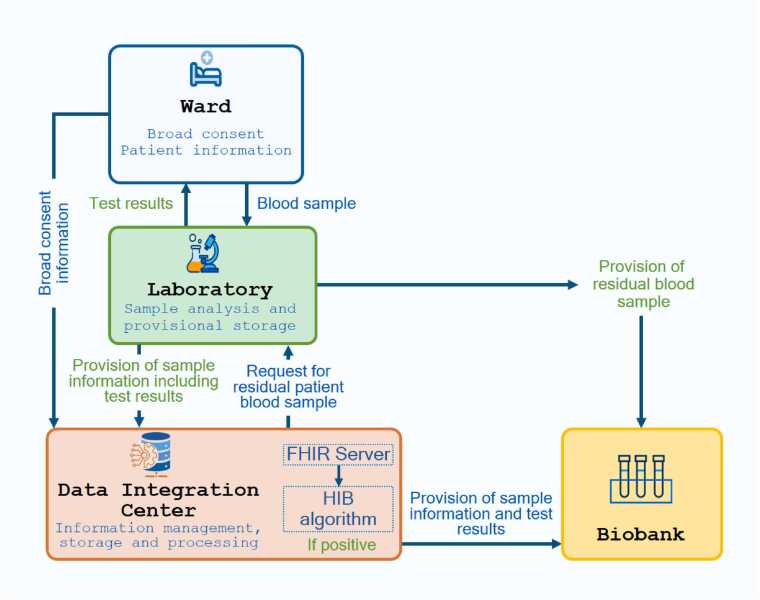
Conceptualization of a healthcare-integrated biobanking (HIB) embedded in a routine hospital workflow management. The algorithms developed in this study identify relevant samples based on available sample information and initiate the biobanking process. FHIR = Fast Healthcare Interoperability Resources.

We developed a general framework (Fig. 1) for the integration of HIB phenotyping algorithms at the Jena University Hospital (JUH). The general framework describes the necessary data workflow to enable biobanking of surplus samples from patients (with advanced CKD in our use case). Starting with the participating wards or the central admission department, patients were contacted to give their written, informed Broad Consent, which, in addition to the use of clinical data, also includes the possible consent to the collection of biosamples. The information on the given informed Broad Consent is transmitted to the data integration center (DIC) [14] as well as the Integrated Biobank Jena (IBBJ) of JUH. DIC is a specialized unit within JUH that promotes secondary use of healthcare and research data to support medical research and improve patient care while ensuring data privacy and security. IBBJ is the accredited (DIN EN ISO 202387) central biobanking facility of JUH, that allows health integrated biobanking of high qualitative biosamples. To enable secondary data use, DIC integrate EHR data from different clinical information systems and transform them into (HL7®) Fast Healthcare Interoperability (FHIR®) [15].

The present study includes 2665 patients (cases with given informed Broad Consent) consented during pilot phase from 09/2020–11/2024 at the JUH [16], [17]. During this phase, the Broad Consent was rolled out to patients at wards of the Department of Internal Medicine IV (Gastroenterology, Hepatology, Infectiology), the Department of Internal Medicine II (Hematology and Oncology) and the Department of General Visceral and Vascular Surgery.

From this Broad Consent cohort, we constructed two analytical cohorts based on different approaches to CKD determination:

(1) A gold-standard cohort of 162 patients with CKD status determined by comprehensive physician chart review
(2) A silver-standard cohort of 1075 patients with CKD status determined by the validated Random Forest algorithm from Weber et al. [13]

Weber et al. [13] used EHRs from 785 patients who had experienced an index hospital stay between 01/2010 and 12/2015 at the JUH [18]. It is important to note that the cases from Weber et al. are completely independent from those reported here even though both cohorts were derived from the same institution (JUH): The covered time frames are non-overlapping, with Weber et al. covering cases from 2010 to 2015 and our validation cohort covering cases from 2018 to 2020. In addition, the cohort described by Weber et al. included only patients who were already deceased at the time the cohort was created [18].

We applied both gold-standard and silver-standard criteria to define the true CKD status. The gold-standard represents the resource-intensive identification of the CKD status by physician chart review of the EHRs. This comprehensive review of each patient’s medical history also included eGFR values from previous hospital or outpatient visits.

**Fig. 2:**
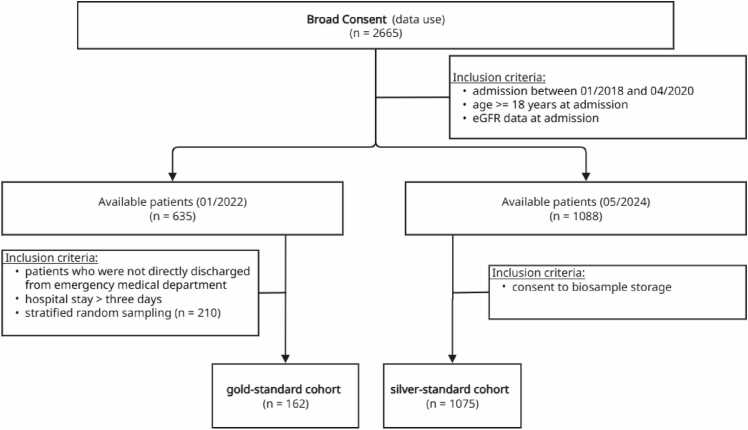
Flowchart of patient selection and cohortst allocation. Patients from the broad consent registry (n = 2665) were screened using defined inclusion and exclusion criteria, resulting in allocation to gold-standard (n = 162) and silver-standard (n = 1075) cohorts. eGFR, estimated glomerular filtration rate.

The gold-standard cohort was derived from the total cohort by matching it to the comorbidity profile of the Weber cohort, resulting in 210 patients. Due to limited physician capacity for manual review, 48 patients were randomly excluded, leaving 162 patients for the final analysis (Fig. 2). Silver-standard cohort relies on a (independently) validated best-performing Random Forest algorithm from Weber et al. to define the “true” CKD status (details see Fig. 2, Table 2). This algorithm includes predictor values from index as well as previous hospital stays (Table 3). As the Random Forest phenotyping algorithms also relies on information not available for the HIB setting, its application resembles the use in retrospective epidemiological studies / registries. We provided both gold-standard and silver-standard criteria as the former may provide better data for a smaller sample whereas the later overcomes resource limitations allowing to report performance measures of the phenotyping algorithms for a larger cohort. The gold-standard cohort is completely included in the silver-standard cohort, except for 13 patients who did not consent to biosample storage.

**Table 2:** Inclusion criteria and cohort characteristics from Weber at al. [13] compared to these reported for HIB phenotyping algorithms validation cohorts (gold-standard and silver-standard cohort) for advanced Chronic Kidney Disease (CKD) phenotyping; HIB: Healthcare Integrated Biobanking.

Criterion	Weber et al.	Validation cohorts for HIB algorithms	
		gold-standard cohort	silver-standard cohort
Sample size	785 patients	162 patients	1075 patients
Time period of index hospital stay	01/2010 and 12/2015	01/2018 and 04/2020	01/2018 and 04/2020
Determination of "true" CKD status	Physician chart review	Physician chart review	Validated algorithm from Weber et al.
Inclusion criteria	age > 18 years; minimum hospital stay of three days	age > 18 years; consent to data use; minimum hospital stay of three days	age > 18 years; consent to data use and biosample storage
Exclusion criteria	patients directly discharged from the emergency medical department; missing eGFR	patients directly discharged from the emergency medical department; missing eGFR	missing eGFR

**Table 3:** Overview of investigated phenotyping algorithms(historyCKD for identifying CKD status within silver-standard cohort, admissionHIB and historyHIB) and their required predictor variables including the availability for each predictor variable; *reverted AKI refers to a decrease in creatinine to less than 66 % of the maximum value within 7 days, identifying patients with acute AKIin regression as described in Weber et al.

predictor variable of phenotyping algorithm	availability of variable	history CKD	admission HIB	history HIB
*age*	admission	x	x	x
*gende*r (m/w)	admission	x	x	x
Length of hospital stay	discharge	x		
*eGFR at admission*	admission	x	x	x
*eGFR at discharge*	discharge	x		
*mean eGFR* at index hospital stay	discharge	x		
eGFR-count at index hospital stay	discharge	x		
eGFR-count (< 60 ML/min/1.73 m²) at index hospital stay	discharge	x		
Acute Kidney Injury (AKI) according to KDIGO criteria	discharge	x		
reverted AKI*	discharge	x		
*mean eGFR* before index hospital stay (from 01/2011)	admission	x		x
ratio between all hospitalizations with eGFR measurements and the total number of hospitalizations before index hospital stay	admission	x		
ratio between the total number of eGFR measurements and the number of all hospitalizations with eGFR measurements before index hospital stay	admission	x		
ratio between the number of eGFR measurements (< 60 ML/min) and the number of all hospitalizations with eGFR measurements before index hospital stay	admission	x		
index classifier (maximum eGFR < 60 ML/min/1.73 m²)	discharge	x		

### 2.2 Data preprocessing and derivation of the silver-standard

To reduce heterogeneity due to data preprocessing we applied the same processing steps as described in Weber et al. First, FHIR resources (Patient, Encounter, Condition) were extracted and flattened using fhircrackr [15]. Subsequently, we applied the inclusion and exclusion criteria described in Fig. 2. Next, we selected the first non-missing laboratory observation for each patient. Key clinical indicators, such as creatinine levels, were used to calculate eGFR, providing an estimate of the patients' kidney function.

For all cohorts analyzed in this manuscript, we used the 2009 version of the CKD-EPI equation [19] without applying the race factor, in line with recommendations of the European Federation of Clinical Chemistry and Laboratory Medicine [20].

Weber et al. reported on 15 CKD algorithms with varying predictor variables and information spanning different time frames. We applied a selection of the best-performing algorithms from Weber et al. to our gold-standard cohort: rule-based algorithms, logistic regression (LR) as well as the three distinct machine learning (ML) algorithms Regularized Generalized Linear Model (GLMnet), Random Forests (RF) and Artificial Neural Network (ANN).

### 2.3 Design of HIB algorithms

**Fig. 3:**
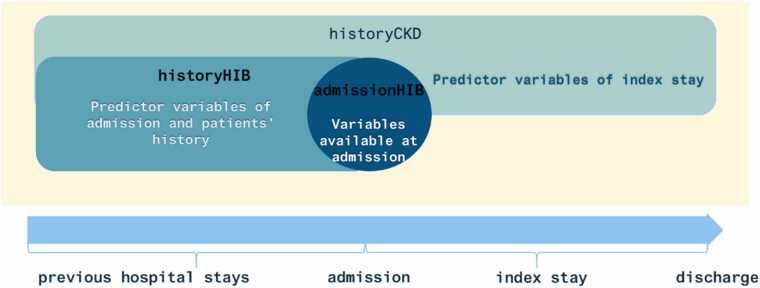
Definition of variable profiles and the time points for algorithms within this study; historyCKD algorithm (including both: predictor variables of index stay as well as variables from previous hospital stays); HIB algorithms: admissionHIB (including predictor variables available at admission) and historyHIB (including predictor variables available at admission and variables from previous hospital stays).

Fig. 3 is a schematic representation of the three different variable profiles reported in this study, one for the CKD algorithm by Weber at al. and two for the HIB algorithms. The historyCKD algorithm for identification of CKD status within silver-standard cohort uses laboratory values (and age/gender) during index hospital stay and from previous hospital stays.

For HIB, early decision-making after a patient's admission is crucial to enable timely, high-quality biobanking of patient samples (Table 3). The variable profiles of the HIB algorithms apply only age, gender and laboratory values (creatinine/GFR) until the first value of hospital admission. Therefore, we distinguish between the following two algorithms:

• *admissionHIB* (without laboratory data from previous hospital stays): age, sex, creatinine at admission
• *historyHIB* (with laboratory data from previous hospital stays until 01/2011): age, sex, creatinine at admission and mean creatinine over previous hospital stays

For the development of HIB algorithms we re-used the data from Weber et al. split randomly into training (80 %) and testing (20 %) sets. A logistic regression model was constructed using the above-mentioned variables for admissionHIB and historyHIB.

For the construction of the HIB algorithms, we used only few parameters due to the early decision-making constraint. In such a low-dimensional setting, logistic regression is known to be robust and to perform comparably to other machine learning methods. We confirmed this by additionally training an RF-HIB algorithm in the Weber cohort, which yielded similar results (supplementary material - STable 1). Considering the need for seamless integration into the hospital information system, we therefore opted for the logistic regression model.

### 2.4 Statistical analysis

Algorithm performance was evaluated using a comprehensive set of measures to assess prediction accuracy and reliability. We calculated sensitivity (recall), specificity, positive predictive value (precision), negative predictive value (NPV), F1-score and accuracy (Acc) to assess the phenotyping algorithms’ ability to correctly classify both positive and negative cases in a binary classification task (presence or absence of gold- or silver-standard CKD diagnosis in the independent 1088 data set that was never used for algorithm development).

To examine the precision-recall across different decision thresholds, we generated Precision-Recall (PR) plots. These visualizations illustrate how the model performance varies as classification thresholds change. The area under the PR curve (AUPR) provides a summary measure of overall performance.

We also generated Receiver Operating Characteristic (ROC) curves, which plots sensitivity (true positive rate) against 1 - specificity (false positive rate) across various thresholds. The area under the ROC curve (AUROC) quantifies the model's discriminative ability between classes. For determining the optimal classification threshold, we employed the Youden Index, which maximizes the sum of sensitivity and specificity.

All statistical analyses were performed using R version 4.3.0 with the following packages: caret and glmnet for model training and evaluation, pROC for ROC analysis, PRROC for precision-recall curves, and ggplot2 for visualization.

## 3 Results

### 3.1 Development and testing of HIB algorithms for CKD identification

We developed and tested two HIB algorithms for CKD identification based on age, gender and laboratory values available at hospital admission using the study cohort described in Weber et al.

**Fig. 4:**
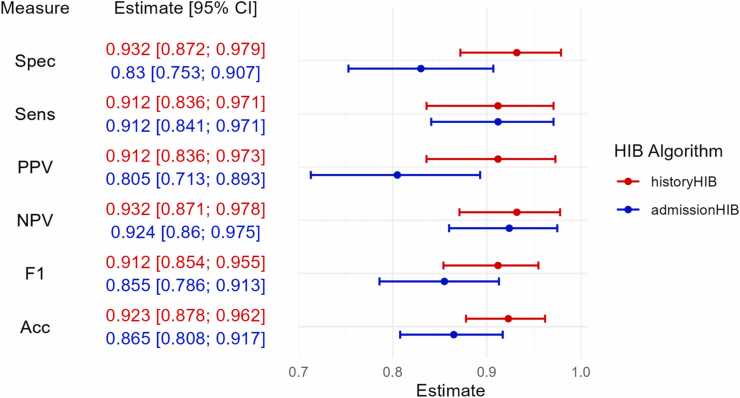
Forest plot - Performance comparison of both new HIB algorithms (red: historyHIB and blue: admissionHIB) across specificity (Spec), sensitivity (Sens), positive predictive value (PPV), negative predictive value (NPV), F1-score and accuracy (Acc) in the test data cohort of Weber et al.; Points indicate estimates and horizontal bars represent 95 % confidence intervals; Cutoff-values (derived from the training cohort of Weber et al.): 0.586 (historyHIB) and 0.478 (admissionHIB).

Among the two approaches (Fig. 4), the historyHIB algorithm, which incorporates laboratory values from previous hospital stays demonstrated superior diagnostic overall performance compared to the admissionHIB algorithm, which is restricted to data from the index admission episode alone (F1-Score 91.2 % for historyHIB vs 85.5 % for admissionHIB). While both algorithms achieved comparable sensitivity (91.2 %) and negative predictive values (NPV: 93.2 % for historyHIB and 92.4 % for admissionHIB), the admissionHIB algorithm exhibited lower specificity (83.0 % vs. 93.2 %) and a reduced positive predictive value (PPV: 80.5 % vs. 91.1 %) when compared to the historyHIB algorithm.

### 3.2 Establishment of a gold- and silver-standard validation cohort

The gold-standard cohort had a younger age profile and overall better health status compared to the cohort described by Weber et al., along with a lower prevalence of CKD (Table 4).

**Table 4:** Characteristics of the gold-standard validation cohort, chronic kidney disease (CKD) was established through manual review of electronic health data (EHR).

Characteristic	gold-standard validation cohort		
	Cohort	CKD	noCKD
n	162	29 (18 %)	133 (82 %)
Age (Years), mean [SD]	62.8 [12.9]	70.8 [9.5]	61.1 [12.9]
Male Gender, n (%)	106 (65.4 %)	17 (58.6 %)	89 (66.9 %)
eGFR at admission (ML/min/1.73 m^2^), median [quartiles]	79.4 [56.4–94.8]	34.4 [18.8–43]	84.1 [70.8–97.2]
Cardiovascular disease, n (%) (myocardial infarction, congestive heart failure, peripheral vascular disease, cerebrovascular disease)	80 (49 %)	23 (79 %)	57 (43 %)
Diabetes mellitus, n (%) (with and without chronic complication)	60 (37 %)	13 (45 %)	47 (35 %)
Liver disease n (%) (mild, moderate or severe)	50 (31 %)	11 (38 %)	39 (29 %)
Malignancy, n (%) (any malignancy, metastatic, solid tumor)	34 (21 %)	2 (7 %)	32 (24 %)

In the gold-standard cohort, we evaluated rule-based algorithms for automated CKD identification previously developed and described by Weber et al. Classification based on discharge summaries, ICD-10 codes, and laboratory thresholds showed varying sensitivity and specificity profiles (Table 5). Laboratory values yielded high specificity but moderate sensitivity, while ICD-10 codes and discharge summaries achieved higher sensitivity with lower specificity. Laboratory values showed the highest overall accuracy (93 %). None of the three rule-based approaches reached an F1-score above 81 %.

**Table 5:** Rule-based classification performance measures for the gold-standard cohort compared to the cohort from Weber et al. (results published there); including sensitivity (Sens), specificity (Spec), positive predictive value (PPV), negative predictive value (NPV), F1-score (F1), accuracy (Acc).

	gold-standard cohort						Weber et al.	
Rule-based classifier	Sens	Spec	PPV	NPV	F1	Acc	F1	Acc
eGFR < 60 ML/min/1.73 m^2^ during Index hospital stay	0.793	0.962	0.821	0.955	0.807	0.932	0.860	0.870
discharge summaries	0.862	0.684	0.373	0.958	0.521	0.716	0.810	0.810
ICD−10 billing codes	0.931	0.872	0.614	0.983	0.740	0.883	0.780	0.820

We also evaluated machine learning (ML)-based algorithms for CKD identification previously developed and tested by Weber et al. Despite epidemiological differences between the gold-standard cohort and Weber’s cohort, the ML algorithms demonstrated consistent performance. This stability indicates the validity and generalizability of these ML models across different study populations, supporting their suitability for generating a silver-standard CKD classification cohort (Table 6). Results for the remaining algorithms can be found in the supplementary material (STable 2, STable 3).

**Table 6:** Results of gold-standard cohort compared to Weber at al. applying Logistic Regression (LR), Regularized Generalized Linear Model (GLMnet), Random Forest (RF) and Artificial Neural Network (ANN) in CKD patients applying the historyCKD algorithm (Predictor variables of index stay and patient’s history).

ML method	gold-standard cohort						Weber at al.	
	sens	spec	PPV	NPV	F1	Acc	F1	Acc
LR	0.667	1.000	1.000	0.927	0.800	0.937	0.896	0.910
GLMNet	0.704	0.991	0.950	0.934	0.809	0.937	0.892	0.904
RF	0.926	0.983	0.926	0.983	0.926	0.972	0.909	0.917
ANN	0.963	0.974	0.897	0.991	0.929	0.972	0.900	0.910

Random Forest (RF) and Artificial Neural Network (ANN) algorithms demonstrated comparable overall F1-scores. The RF algorithm (historyCKD algorithm - Predictor variables of index stay and patient’s history) was selected for CKD identification in the silver-standard cohort due to its slightly higher specificity and positive predictive value (PPV). Both metrics are considered especially important for HIB (Table 1).

To evaluate the agreement between the CKD identification in gold-standard and silver-standard cohort in the overlapping 71 cases, we performed a detailed analysis, including a confusion matrix and the calculation of agreement metrics. The results showed a high degree of consistency with an accuracy of 97 % and a Cohen's kappa value of 0.91, indicating high agreement. These results demonstrate the reliability of the Random Forest-based silver-standard despite its inherent dependence on a single algorithm and its potential biases.

Within the silver-standard cohort, the ML-based classification estimated a CKD prevalence of 10 %. Consistent with observations in the gold-standard cohort, individuals classified as having CKD were older and presented with a higher comorbidity burden (Table 7).

**Table 7:** Characteristics of the silver-standard cohort with algorithm-based determination of “true” CKD status.

Characteristics	silver-standard cohort		
	cohort	CKD	noCKD
n	1075	108 (10 %)	967 (90 %)
Age (Years), mean [SD]	59.5 [15.3]	69.3 [11.4]	58.4 [15.3]
Male Gender, n (%)	619 (57.6 %)	58 (53.7 %)	561 (58.0 %)
eGFR at admission (ML/min/1.73 m2), median [quartiles]	86.7 [69.3–99.0]	40.8 [31.1 – 46.3]	89.3 [76.3 – 100.2]
Cardiovascular disease, n (%) (myocardial infarction, Congestive heart failure, Peripheral vascular disease, Cerebrovascular disease	190 (18 %)	41 (38 %)	149 (15 %)
Diabetes mellitus, n (%) (with and without chronic complication)	132 (12 %)	32 (30 %)	100 (10 %)
Liver disease n (%) (mild, moderate or severe)	122 (11 %)	14 (13 %)	108 (11 %)
Malignancy n (%), (any malignancy, metastatic, solid tumor)	286 (27 %)	23 (21 %)	263 (27 %)

In the cohort from Weber et al. and in the validation cohorts, there was a similarly strong correlation between age and CKD (silver-standard: r = 0.37, gold-standard: r = 0.43, Weber: r = 0.32), as well as between comorbidities and CKD (silver-standard: r = 0.45, gold-standard: r = 0.41, Weber: r = 0.37).

### 3.3 Validation of HIB algorithms in gold- and silver-standard cohort

For validation, we applied the HIB algorithms to the silver-standard cohort. To evaluate the approach of using a silver-standard for algorithm validation, we additionally applied the algorithms to the gold-standard cohort, which was derived from the same source cohort.

**Fig. 5:**
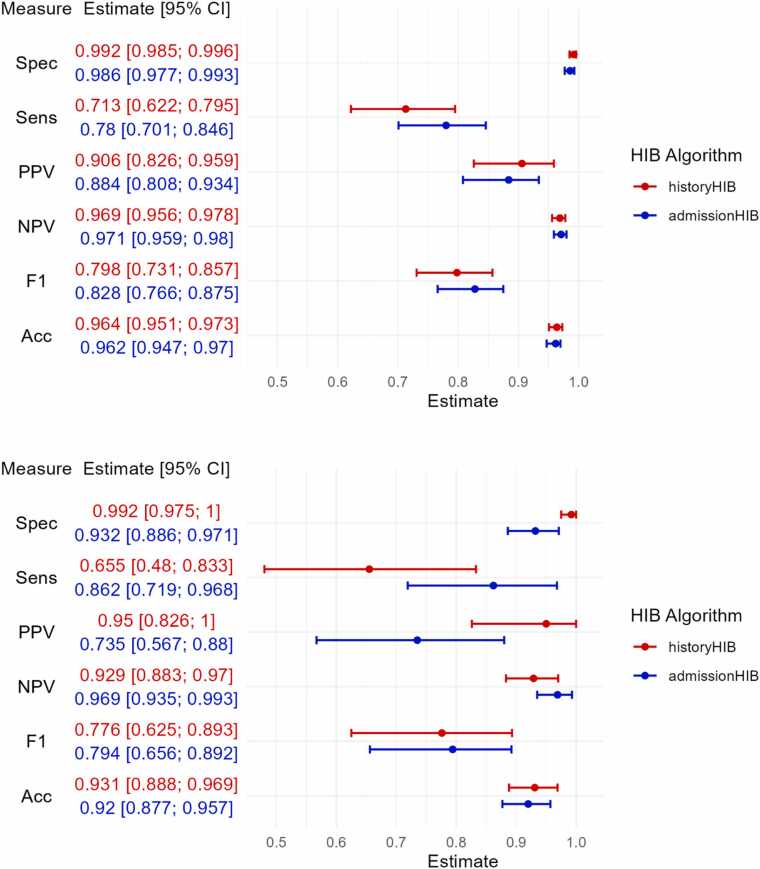
Results of HIB algorithms evaluated in both silver-standard (upper panel) and gold-standard (lower panel) cohort algorithms; including sensitivity (Sens), specificity (Spec), positive predictive value (PPV), negative predictive value (NPV), F1-score (F1), and accuracy (Acc), each with corresponding 95 % confidence intervals (CI) for each metric. Cutoff values were set to 0.586 for historyHIB and 0.478 for admissionHIB, consistent with the Weber et al. cohort.

In the silver-standard cohort, both algorithms demonstrated excellent specificity (99.2 % for historyHIB and 98.6 % for admissionHIB), along with comparable positive predictive values (PPV) of 90.6 % and 88.4 %, respectively. The F1-scores were also high (79.8 % for historyHIB and 82.8 % for admissionHIB), only slightly lower than those in the Weber et al. cohort (Fig. 5).

The results observed in the silver-standard cohort were largely mirrored in the gold-standard cohort. Specificity (93.2 %) and PPV (73.5 %) were lower for the admissionHIB algorithm in the gold-standard cohort compared to the silver-standard cohort albeit differences were largely within the wide confidence intervals in the gold-standard cohort. F1-scores and accuracy were comparable across all algorithms in both cohorts (Fig. 5). Notably, the results obtained from the silver-standard cohort exhibited consistently narrower confidence intervals compared to those obtained from the gold-standard cohort. This difference primarily reflects the larger sample size and greater data stability in the silver-standard cohort, which contributes to more precise estimates. Conversely, the gold-standard cohort’s relatively smaller sample size leads to wider confidence intervals, indicating greater uncertainty in the estimates.

**Fig. 6:**
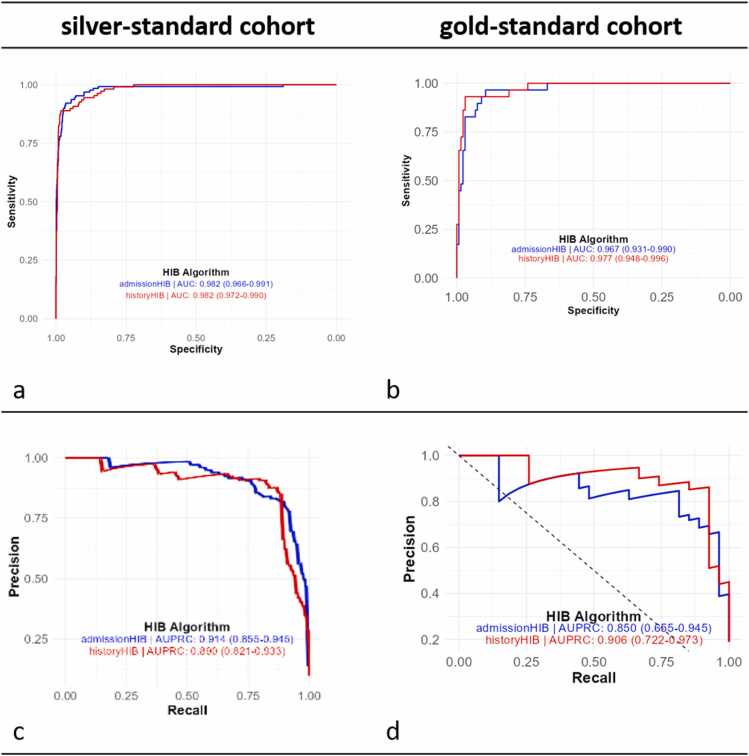
Receiver operating characteristic (ROC, a+b) curves and precision-recall (PR) curves (c+d) for the admissionHIB (blue) and historyHIB (red) algorithms, evaluated using the silver-standard cohort (left) and gold-standard cohort (right).

Consistent with the F1-scores, both the area under the receiver operating characteristic curve (AUC-ROC) and the area under the precision-recall curve (AUPRC) exhibited similarly high values across cohorts. A slightly superior discriminatory performance of the historyHIB algorithm compared to admissionHIB, as measured by AUPRC in the gold-standard cohort, was not observed in the silver-standard cohort (Table 8, Fig. 6). This difference remained largely within the respective confidence intervals, though.

**Table 8:** Performance measures of the HIB algorithms, including the area under the receiver operating characteristic curve (AUROC) and area under the precision-recall curve (AUPRC) for both gold-standard and silver-standard cohort.

**HIB algorithm**	**cohort**	**n**	AUROC	AUPRC
historyHIB	gold-standard	162	0.977 (0.948–0.996)	0.906 (0.722–0.973)
	silver-standard	1075	0.982 (0.966–0.991)	0.890 (0.821–0.933)
admissionHIB	gold-standard	162	0.967 (0.932–0.990)	0.850 (0.655–0.945)
	silver-standard	1075	0.982 (0.966–0.991)	0.914 (0.855–0.945)

## 4 Discussion

This study describes the development of novel CKD identification algorithms specifically tailored for healthcare-integrated biobanking. The algorithms demonstrated robust transferability when applied to cohorts with markedly different disease prevalence, comorbidity profiles, and demographic characteristics compared to the original Weber et al. cohort. Furthermore, the generation of a silver-standard validation cohort was shown to be a valid and practical method for assessing algorithm performance in larger populations.

The performance of the HIB algorithms for CKD in the training cohort is inferior to recently published algorithms for CKD [9], [21], [22]. This could at least partly be explained by the fact that the algorithms from the aforementioned studies require multiple data from previous medical encounters for completion, whereas HIB algorithms in the current study are based either solely on data from the index hospital stay or - if previous history was included - only laboratory data from the same institution. These restrictions reflect the “real-world” conditions of the decentralized German health system in which no comprehensive central registry for patient health records has been completely implemented so far. In addition, compared with the Weber cohort, the CKD prevalence in the validation cohorts is relatively low, at least partly due to the recent rollout of the broad consent process. This lower prevalence may in turn affect the PPV. As patients are followed during future hospital stays, both prevalence and PPV are expected to increase. Nevertheless the collection of false positives cannot be fully avoided, though this does not affect sample collection workflow. For CKD-focused research, a secondary algorithm for the biobanked samples using the full patient dataset can confirm true positives, as shown by our group in a diabetes mellitus use case [23].

For generating the silver-standard, we first tried rule-based algorithms from Weber et al. [13] utilizing laboratory values, ICD-10 billing codes, and/or discharge summaries. However, this revealed only a moderate performance in identifying CKD and results were inferior to those by Weber et al. Possible explanations include the lower CKD prevalence in the validation cohort compared to the development cohort. As large studies and registries often rely only on such rule-based CKD identification tools [21], [24] there is a risk for both false positive and false negative cases. In addition, these results underscore the necessity to also advance algorithms development.

Correspondingly, the ML-based algorithms from Weber et al. demonstrated a very good performance in the gold-standard validation cohort. Since creating a gold-standard through manual review of patient EHRs is time-consuming and impractical for large cohorts, we established a silver-standard based on the decisions of the ML-based algorithm developed by Weber et al. The HIB algorithms performed similarly well in both the gold-standard and silver-standard cohort. The creation of silver-standards using various automated review techniques has been discussed and applied in several studies [25]. Wagholikar et al. [26] describe and validate a rule-based approach for automated review employing ICD-10 codes. However, concerning CKD, we have previously demonstrated - and confirm results in the present study - that reliance on ICD-10 codes underestimates CKD prevalence, potentially resulting in selection bias for the silver-standard. Similarly, Searle et al. [27], using the MIMIC-III cohort, reported the underperformance of ICD-10–based approaches for various diseases, though not specifically for CKD. They proposed and validated an automated review silver-standard incorporating broader EHR data, such as discharge summaries. Consistent with these findings, we previously demonstrated that integrating multiple data sources improves CKD detection accuracy [13].

We also demonstrated similar improvements in performance for the identification of diabetes mellitus when using broad EHR data, highlighting the potential for the improvement of classification accuracy as well as the creation of high quality silver-standards for further disease entities [23].

Here, we show that an approach based on laboratory values offers a robust foundation for constructing a silver-standard cohort.

Although validation for the HIB algorithms is performed in the same hospital and by the same research group, this study also meets criteria for external validation according to de Hond et al. [28]: The first criterion is domain: The validation cohort differs significantly from the dataset used for algorithm development regarding demographics and CKD prevalence. The second criterion is time: The validation was conducted with participants attending the hospital almost ten years later than the patients from the development study. Aside from changes in patient characteristics it is important to note that clinical workflows and proceedings in the laboratory and the biobank changed substantially within that time gap with increased automation and upgraded assays. Finally, the validation cohort is representative of the target population that is hospitalized patients who agreed that their clinical data and surplus biospecimen can be used for research purposes in an anonymized way [29], [30]. Despite the importance of carrying out an external validation, such studies are relatively sparse [4], [29], therefore, the present study fills an important gap.

We believe that this study outlines a roadmap for the further validation and implementation of the HIB CKD algorithm. The next step will be a retrospective and prospective multicenter validation. For the retrospective validation, we plan to leverage the National Portal for Medical Research Data (FDPG), which is currently being established in Germany [31], [32]. In the context of a prospective validation, the ability to integrate the algorithm into existing hospital information system infrastructures represents a key prerequisite for the feasibility of multicenter studies. This consideration further supports our decision to retain a model based on simple logistic regression, as such algorithms are typically easier to implement across diverse hospital environments.

Future evaluations of CKD HIB algorithms should also incorporate a systematic assessment of resource utilization and cost-efficiency within HIB workflows, e.g. as outlined by Rush et al. [33]. Such analyses should ideally be conducted after multi center validation and over a longer observation period, as the current sample size and study duration are insufficient for a robust evaluation.

Beyond CKD, this study may serve as a blueprint for other diseases that could benefit from integration into the HIB framework. The transferability of this approach depends on specific criteria: the disease must be identifiable by routinely measured laboratory parameters obtained at patient admission, allowing for early identification and biobanking of surplus samples. Furthermore, for large-scale validation, the generation of a silver-standard requires an automated algorithm capable of accurately identifying cases. We have recently described an algorithm capable of identifying diabetes mellitus based on laboratory data collected within the first 72 h of hospitalization [23]. Additionally, we showed that automated text analysis of discharge summaries may provide an excellent silver-standard [23]. Regarding more acute diseases, recent studies have demonstrated that urinary tract infections and sepsis can be identified in hospitalized patients using routinely available clinical chemistry parameters [34], [35]. Both studies used ICD-10 coding as ground truth, enabling the creation of large silver-standard datasets.

Finally, it should be pointed out that not all diseases are equally suited for inclusion into HIB workflows outlined in this study. Some conditions rely on highly specialized laboratory markers (e.g., certain malignancies) that are not routinely measured, while others, such as hypertension, lack reliable laboratory indicators altogether. For these cases, alternative strategies will be needed to enable their integration into the HIB process.

## 5 Limitations

While time and patient characteristics of the validation cohorts differed from the Weber et al. cohort , there is no geographical external validation. Therefore, as outlined before, the study results warrant confirmation through a prospective and multicenter approach.

Secondly, the high prevalence of malignancy-associated diagnoses in the validation cohort, reflects pilot-phase recruitment. This over-representation may affect performance, as e.g. acute kidney injury caused by chemotherapy can transiently alter creatinine and eGFR, mimicking CKD. Performance may improve cohorts with a lower malignancy-prevalence.

Several further methodological limitations warrant careful consideration. While this silver-standard methodology addresses the challenge of limited sample size, it introduces variability in performance evaluation. Comparative analysis of individual performance measures, such as sensitivity, specificity and PPV, reveals acceptable consistency between gold- and silver-standard assessments within the limits of uncertainty. Finally, for the novel HIB algorithms, we employed a simple approach using logistic regression. Considering the limited number of variables and the preference of simplicity, logistic regression appeared to be the appropriate choice. More complex algorithmic approaches would likely present implementation challenges in clinical practice without a substantial likelihood of significantly enhancing performance.

## 6 Conclusions

In this study, we successfully developed algorithms tailored to the need of HIB and we demonstrate that a silver-standard can be utilized to robustly validate the overall performance of the algorithms in larger cohorts. Future research should aim at the prospective multi center validation of the HIB-CKD algorithm and the further automation of HIB-related workflows. Beyond CKD, the proposed framework holds potential for application to other diseases identifiable through routinely measured laboratory parameters.

## Compliance with ethical standards

This study was conducted in accordance with the Declaration of Helsinki (as revised in 2013) and was approved by the local ethics committee of the Friedrich Schiller University of Jena at the Faculty of Medicine (Ethik-Kommission der Friedrich-Schiller-Universität Jena, Bachstraße 18, 07740 Jena, Germany, ethikkommission@med.uni-jena.de, #4639–12/15 & 2023–3148).

## Funding

This work was supported by the Deutsche Forschungsgemeinschaft (DFG) under grant KI 564/2–1 within the STAKI2B2 project (“Semantic Text Analysis for Quality-controlled Extraction of Clinical Phenotype Information within the Framework of Healthcare-Integrated Biobanking”); Federal Ministry of Research, Technology and Space (BMFTR) within the LPI project by funding program: quantum technologies – from basic research to market (contract number: 13N15711); the SMITH consortium and the Jena University Hospital – Friedrich Schiller University Jena is supported by the German Federal Ministry of Education and Research (01ZZ1803C).

## CRediT authorship contribution statement

**Boris Betz:** Conceptualization, Formal analysis, Investigation, Methodology, Supervision, Validation, Visualization, Writing – original draft, Writing – review & editing. **Claudia Fischer:** Conceptualization, Data curation, Formal analysis, Investigation, Methodology, Software, Validation, Visualization, Writing – original draft, Writing – review & editing. **Johannes Stolp:** Formal analysis, Investigation, Visualization, Writing – review & editing. **André Scherag:** Conceptualization, Funding acquisition, Methodology, Project administration, Resources, Supervision, Writing – review & editing. **Danny Ammon:** Resources, Writing – review & editing. **Michael Kiehntopf:** Conceptualization, Funding acquisition, Investigation, Methodology, Project administration, Resources, Supervision, Writing – review & editing.

## Declaration of Competing Interest

All authors have reviewed the manuscript and confirm that there are no financial, personal, or professional relationships that could be perceived as influencing the objectivity of this research. No author has received funding, compensation, or any other benefits from organizations or entities that could be considered to create a conflict of interest with regard to the subject matter of this publication.

## Data Availability

The data supporting the results of this study are subject to restrictions due to the Thuringian Hospital Act and are therefore not publicly available. The data are available from the author (C.F.) on reasoned request and with the approval of the local ethics committee of the Friedrich Schiller University of Jena at the Faculty of Medicine (Ethik-Kommission der Friedrich-Schiller-Universität Jena, Bachstraße 18, 07740 Jena, Germany, ethikkommission@med.uni-jena.de) and the data protection officer of Jena University Hospital (Zentrum für Gesundheits- und Sicherheitsmanagement, Beauftragte für Datenschutz, Am Klinikum 1, 07747 Jena, Germany, Datenschutzbeauftragter@med.uni-jena.de).
