# Supplementary material for: Design and validation of an automated healthcare-integrated biobanking algorithm for identification of advanced chronic kidney disease
Source: Comput Struct Biotechnol J. 2025 Nov 2;28:487–97. doi: 10.1016/j.csbj.2025.10.061 (PMC12657308; doi:10.1016/j.csbj.2025.10.061)
Supplement: Supplementary file 1 — Supplementary material [file mmc1.docx]

### Supplementary

STable 1: Results of training an RF-HIB algorithm (admissionHIB and historyHIB) in the Weber cohort compared to the logistic regression, we used in this study.

| **Model** | **HIB algorithm** | **sens** | **spec** | **PPV** | **NPV** | **F1** | **Acc** |
| --- | --- | --- | --- | --- | --- | --- | --- |
| LR | admissionHIB | 0.912 | 0.830 | 0.805 | 0.924 | 0.855 | 0.865 |
|  | historyHIB | 0.912 | 0.932 | 0.912 | 0.932 | 0.912 | 0.923 |
| RF | admissionHIB | 0.779 | 0.807 | 0.757 | 0.826 | 0.768 | 0.795 |
|  | historyHIB | 0.926 | 0.841 | 0.818 | 0.937 | 0.869 | 0.878 |

Results of validation study compared to Weber at al. applying Logistic Regression (LR), Generalized Linear Model (GLMnet), Random Forest (RF) and Artificial Neural Network (ANN) in CKD patients:

STable 2: indexCKD algorithm (Predictor variables of index stay)

| **ML method** | **validation study** | | | | | | **Weber at al.** | |
| --- | --- | --- | --- | --- | --- | --- | --- | --- |
|  | **sens** | **spec** | **PPV** | **NPV** | **F1** | **Acc** | **F1** | **Acc** |
| **LR** | 0.963 | 0.957 | 0.839 | 0.991 | 0.897 | 0.958 | 0.900 | 0.910 |
| **GLMNet** | 1.000 | 0.939 | 0.794 | 1.000 | 0.885 | 0.951 | 0.894 | 0.904 |
| **RF** | 0.963 | 0.957 | 0.839 | 0.991 | 0.897 | 0.958 | 0.894 | 0.904 |
| **ANN** | 1.000 | 0.930 | 0.771 | 1.000 | 0.871 | 0.944 | 0.900 | 0.910 |

STable 3: historyCKD algorithm (Predictor variables of index stay and patient’s history)

| **ML method** | **validation study** | | | | | | **Weber at al.** | |
| --- | --- | --- | --- | --- | --- | --- | --- | --- |
|  | **sens** | **spec** | **PPV** | **NPV** | **F1** | **Acc** | **F1** | **Acc** |
| **LR** | 0.667 | 1.000 | 1.000 | 0.927 | 0.800 | 0.937 | 0.896 | 0.910 |
| **GLMNet** | 0.704 | 0.991 | 0.950 | 0.934 | 0.809 | 0.937 | 0.892 | 0.904 |
| **RF** | 0.926 | 0.983 | 0.926 | 0.983 | 0.926 | 0.972 | 0.909 | 0.917 |
| **ANN** | 0.963 | 0.974 | 0.897 | 0.991 | 0.929 | 0.972 | 0.900 | 0.910 |
